# Part–whole effects in visual number estimation

**DOI:** 10.3758/s13414-025-03158-8

**Published:** 2026-01-08

**Authors:** Chenxiao Guan, David Schwitzgebel, Chaz Firestone, Alon Hafri

**Affiliations:** 1 Department of Psychology and Behavioral Sciences, Zhejiang University, Hangzhou, 310058 China; 2 Department of Psychological and Brain Sciences, Johns Hopkins University, Baltimore, MD 21218 USA; 3 Institut Jean Nicod/Laboratoire de Sciences Cognitives et Psycholinguistique, École Normale Supérieure–⁠Paris Sciences et Lettres, 75005 Paris, France; 4 Department of Linguistics and Cognitive Science, University of Delaware, 15 Orchard Road, Ewing Hall, Room 417, Newark, DE 19716 USA

**Keywords:** Approximate number system, ANS, Perception, Shape, Possibility, Visual relations

## Abstract

In a single glance at a collection of objects, we can appreciate their numerosity. But what are the “objects” over which this number sense operates? Most work in this domain has implicitly assumed that we estimate the number of discrete, bounded individuals actually present in the visual field. However, in many instances we can construe such individuals as potential *parts* of composite objects that they can create—as when we assemble furniture or complete a jigsaw puzzle. Here, we demonstrate that visual numerosity estimation is sensitive to such part–whole relations, such that the number of items in a display is underestimated when it contains spatially separated but easily combinable objects. Participants saw brief displays containing noncontiguous “puzzle-piece” stimuli, and reported which display had more pieces. Crucially, most of the pieces appeared in pairs that either could or could not efficiently combine into new objects. In four experiments, displays with combinable pieces were judged as less numerous than displays with noncombinable pieces—as if the mind treated two geometrically compatible pieces as being the single whole object they could create. These effects went beyond various low-level factors, and they persisted even when participants were explicitly trained to treat individual pieces as the units that should be counted. Thus, despite the many ways that sets of objects may be construed for the purposes of counting, visual perception automatically takes into account the ways that object parts may combine into wholes when extracting numerosity from visual displays.

## Introduction

Daily life often requires us to represent how many items are in view, as when children decide how many toys their friend has or adults determine whether they have gathered enough vegetables for dinner. Crucially, answering such questions involves first and foremost *estimating* the number of relevant items.

Adult humans have a remarkable capacity to represent exact quantities using cognitive machinery, but they also share another capacity with both prelinguistic infants and nonhuman animals: the ability to approximate numerical quantities. Many researchers posit that these representations arise through a suite of cognitive mechanisms often called the Approximate Number System (ANS). This system emerges early on (Izard et al., 2008, 2014; Xu & Spelke, 2000) and persists into adulthood, as explored in studies requiring observers to estimate the number of dots in a briefly viewed display (Barth et al., 2003; Cordes et al., 2001). Indeed, this process is rapid and automatic (Halberda & Feigenson, 2008; Sanford et al., 2023), and may even exhibit one of the key signatures of visual processing: retinotopic adaptation (Burr & Ross, 2008; see Yousif et al., 2024, and Myers et al., 2025, for discussion and evaluation of alternative explanations).

Numerous aspects of the ANS remain vigorously debated (see, e.g., Clarke & Beck, 2021, and commentaries found within). For example, some researchers argue that estimates of number might arise via a nonnumerical sensory integration system that integrates continuous perceptual variables correlated with numerosity rather than relying on an ANS (Gebuis et al., 2016; Gebuis & Reynvoet, 2012). Related research argues for a “sense of magnitude” rather than “sense of number” (Leibovitch et al., 2017).

**Fig. 1:**
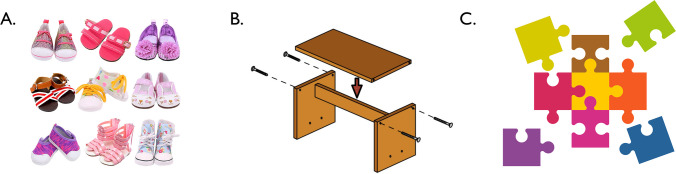
Displays with ambiguous numbers of items. **A.** An array of shoes (nine pairs or 18 individuals). **B.** A diagram for assembling a table (depicting one full table, two parts, or four pieces of wood). **C.** A set of puzzle pieces that can make a complete puzzle (one nearly completed puzzle, five disconnected elements, or nine individual pieces). (Color figure online)

However, with regard to visual number estimation, one surprisingly fundamental question has remained underexplored—namely, what counts as an item to be enumerated in the first place? Consider Fig. 1A. When one is determining their quantity of shoes, one must first decide whether to count individual shoes or pairs. Consider also Figs. 1B and 1C: These images contain discrete, segmented individuals, but it is clear that these individuals can combine with one another, potentially becoming part of a larger composite object. These observations lead to a question: How, if at all, does visual number estimation accommodate the relationship between individual object parts and the wholes they can create?

### Segmented objects as “units” of visual number estimation

Many visual processes, including attention and working memory, are at least partly “object-based.” For example, in studies of visual attention, observers are faster to identify targets when they appear on the same object as a probe rather than on a different (equidistant) object (Egly et al., 1994; see also Kahneman et al., 1992). Likewise, the capacity of visual working memory depends on whether to-be-remembered features appear within the same object or on different objects (e.g., Luck & Vogel, 1997); and even high-level social relations like *chasing* appear to be object-based (van Buren et al., 2017).

This aspect of visual processing also arises in number estimation, which appears to rely on discrete, segmented objects. Perhaps most strikingly, Franconeri et al. (2009) asked observers to estimate which of two displays had more items. In one display, items were connected by thin lines, and in the other, they were not. Remarkably, observers underestimated the number of items in the connected-item display, despite being told to ignore the connecting lines (see also He et al., 2009; Kirjakovski & Matsumoto, 2016). Relatedly, a study by Adriano et al. (2021) found that observers systematically underestimated the numerosity of displays containing *illusory* connectors—lines defined only by aligned contours on the items themselves (similar to Kanizsa triangles), rather than by luminance discontinuities between them (Grossberg & Mingolla, 1987). These sets of findings suggest that linking objects together (whether through actual physical connections or perceptual inference via modal completion) can cause the mind to underestimate the number of objects present.

### Object parts and the wholes they can create

Notably, all of the above cases (including the object-based numerosity studies) assume, either implicitly or explicitly, that the input units to numerical estimation are *actual* objects—discrete, segmented individuals appearing in the visual field—even when those objects are composed of items linked by physical connections (whether actual or illusory; Adriano et al., 2021; Franconeri et al., 2009). But consider again Figs. 1B and 1C. Although we may appreciate the spatially distinct parts of the table in Fig.1B, or the individual puzzle pieces in Fig. 1C, we can also appreciate the full table or completed puzzle that such arrangements suggest.

In recent work, we demonstrated that visual perception automatically represents the potential objects that combinable object parts may create (Guan & Firestone, 2020). In that study, participants were instructed to respond, under time pressure, to a particular target (e.g., a square) appearing within a stream of distractors (broken-up square “parts”). Sometimes, the distractors were pairs of objects that could create the target in combination; in other cases, the distractors could not combine to create the target but shared other low-level properties (e.g., shape, color). Strikingly, participants occasionally “confused” combinable objects for their potential wholes, as indicated by a greater number of false alarms to pairs of distractors that could create their target than to pairs that could not. In other words, participants represented combinable object parts *as* their combined wholes. In this paper, we will focus on this specific phenomenon: the propensity for separate parts to be perceptually combined into a single, coherent object.

### The current study: Do potential objects “count” too?

**Fig. 2:**
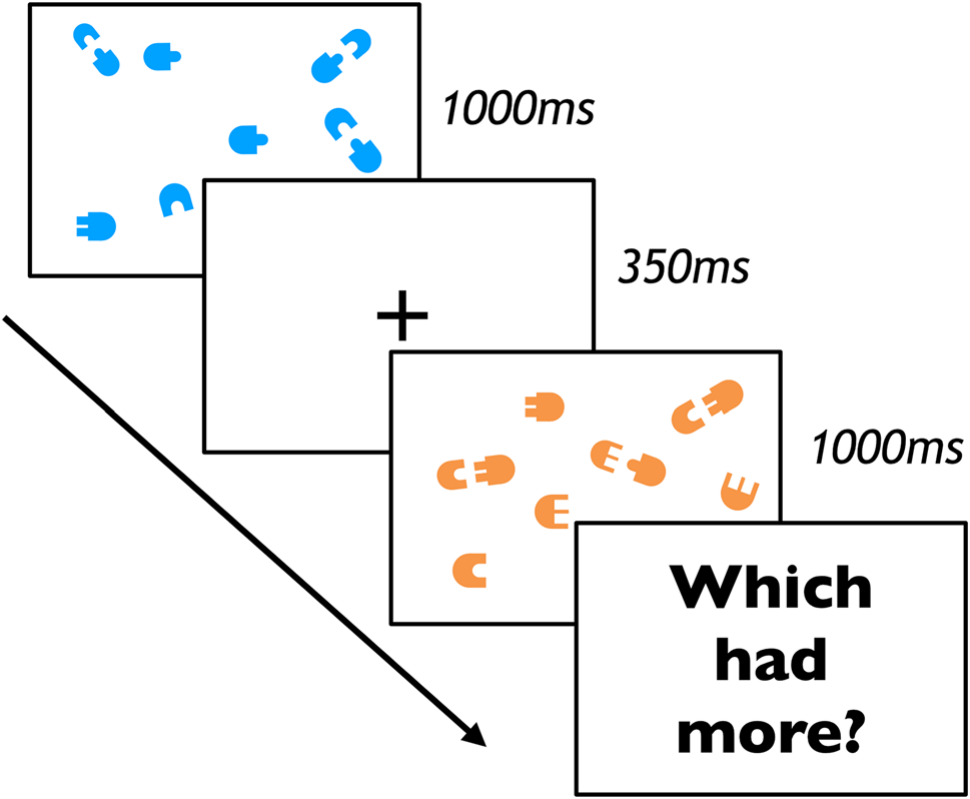
Experimental design for Experiments 1–4. On each trial, participants performed a numerosity comparison task. They observed two displays of stimuli (one blue, one orange), separated by a fixation cross. Each display contained a certain number of “puzzle-piece” stimuli. After seeing both displays, participants were asked which of the displays had more pieces (or fewer pieces, for a separate group of participants). Crucially, in one display the pieces were in a “matched” configuration (in which the pieces could efficiently combine into whole objects), and in the other display, pieces were in a “mismatched” configuration (in which the pieces could not, Experiments 1–3) or in an outward-facing configuration (in which the matching pieces faced outward so could not efficiently combine, Experiment 4). (Color figure online)

Might such potential wholes be treated by the visual system as input units for other visual processes, like visual number estimation and comparison? In the current study, we test this possibility. Participants were presented with brief displays of “puzzle-piece” stimuli, and were simply asked to decide which display had more pieces (Fig. 2). One display contained pairs of pieces that could efficiently combine into new objects, and the other display contained pairs that could not. In Experiment 1, pieces with protrusions appeared adjacent to pieces with matched indentations (so they could combine into a single whole object) or mismatched indentations (so they could not). Experiment 2 replicated this design with additional instructions and training which ensured that participants understood that they should consider individual puzzle pieces as the unit of comparison between displays. In Experiment 3, pairs of pieces were vertically offset from one another in order to minimize low-level visual grouping cues such as modal completion. In Experiment 4, pairs of pieces with matching protrusions and indentations faced toward one another (so they could easily combine into a single whole object) or faced away from one another (so they could not). To foreshadow the key result, in all of our experiments, participants judged displays with combinable objects as less numerous than displays with objects that were not combinable, suggesting that visual comparison of numerosities takes into account the (potential) whole objects that can be made of (actual) visible parts. Demos of these experiments can be viewed at https://www.palresearch.org/partwholenumber.

**Fig. 3:**
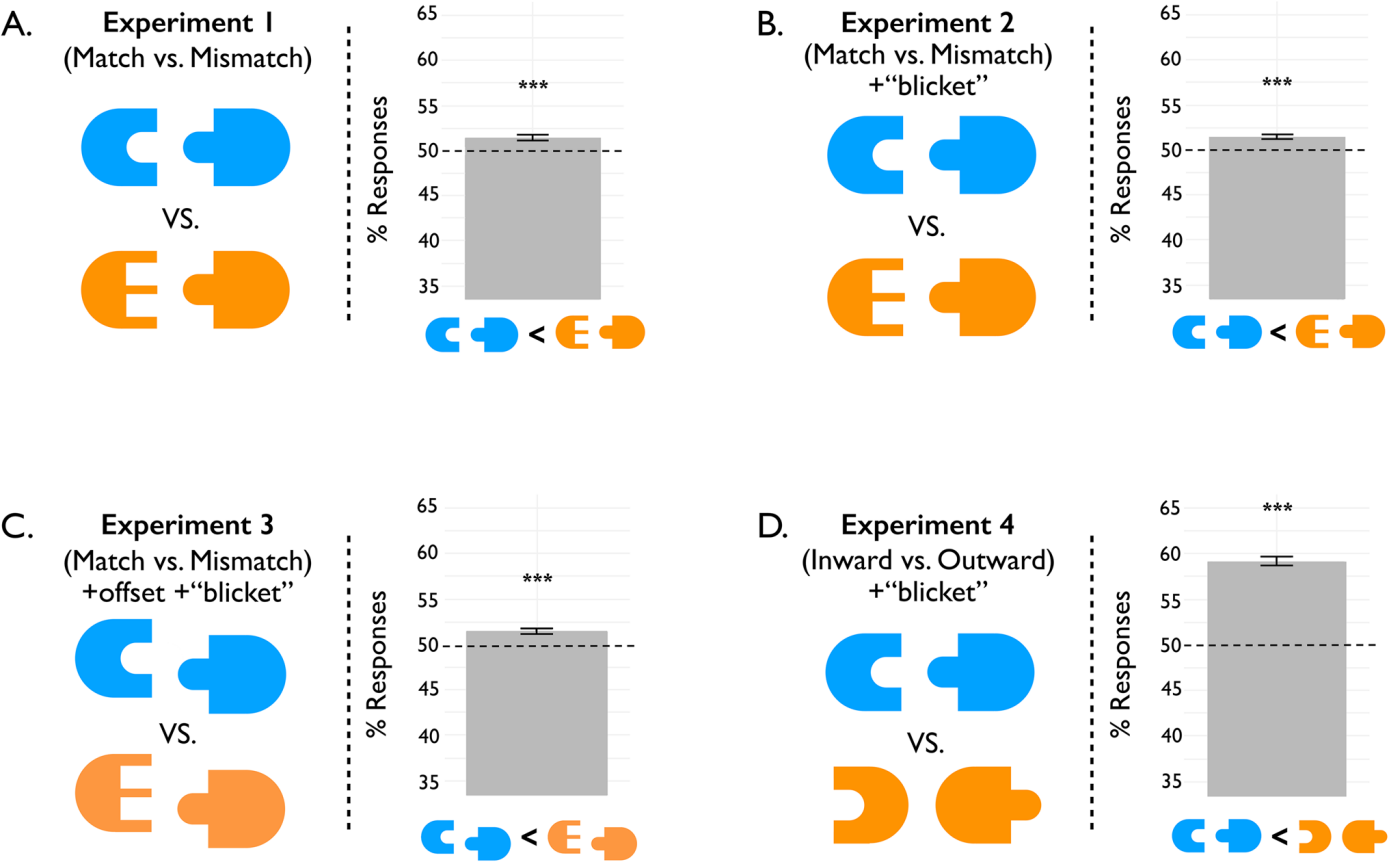
Display conditions and results for Experiments 1–4, collapsed across numerosity ratios. In Experiments 1–3, one display had “matched” pieces (that could combine into a whole object) and one display had “mismatched” pieces (that could not). Experiment 4 featured only matching pieces, but they faced inward in one display and outward in the other. Experiments 2–4 also ensured that participants understood the instruction to judge the number of puzzle pieces (and not their potential wholes) by including a pretask in which they were instructed to count the “blickets” (puzzle pieces) with feedback. Bars show the mean proportion of responses where matched (or inward) displays were chosen as less numerous. In all four experiments, the results show that participants reported matched displays (or inward displays) as less numerous than mismatched (or outward) displays. Error bars reflect standard errors of the means. *** *p* <.001. (Color figure online)

## Experiment 1: Combinable parts as single wholes in number estimation

Can the combinability of distinct object parts into wholes influence visual number estimation? Experiment 1 tested this possibility using a standard two-interval numerosity comparison task, with displays containing either combinable or noncombinable "puzzle-piece" objects.

### Methods

#### Open practices

All experiment materials, including preregistrations, stimuli, code, analyses, and anonymized data, are available at: https://www.palresearch.org/partwholenumber. This website also includes demos of each experiment, so readers can experience the tasks for themselves.

#### Participants

Two-hundred participants were recruited from Prolific. (For a discussion of the reliability of this subject pool, see Peer et al., 2017.) This sample size was determined from pilot studies. Sample sizes were preregistered for this and all other experiments. To ensure the quality of our data, participants were prescreened to be US nationals, to have completed a minimum of 50 previous submissions, to have achieved a minimum approval rate of 85% (on their previous Prolific submissions), and to have not participated in other related experiments (e.g., pilots).

#### Stimuli and procedure

Stimuli consisted of “puzzle-piece” objects: modified half-ovals with indentations or protrusions. These indentations and protrusions were either square or round, and individual pieces either had one or two indentations or protrusions. Stimuli were grouped into two categories: “singles” and “pairs.” Singles consisted of individual pieces, whereas pairs consisted of two pieces facing each other, one of which had indentation(s) and one of which had protrusion(s). Pairs were grouped into two subcategories: “matched” and “mismatched” (Fig. 3A). Matched pairs consisted of combinable pieces—the protrusions could fit into the indentations. Therefore, it was easy to perceptually “combine” the pieces into a complete, unbroken object (Guan & Firestone, 2020). By contrast, mismatched pairs consisted of pieces that could not easily combine into one object. Although the mismatched pieces faced toward one another, the protrusions could *not* fit into the indentations (e.g., a single square protrusion facing two round indentations).

On each trial of the study, participants viewed two displays of puzzle pieces presented sequentially, each of which was presented for 1,000 ms (Fig. 2). The displays (height: 500 px; width: 700 px) were separated by a fixation cross lasting for 350 ms. After these displays, participants were asked to determine which of the two displays had more pieces (or fewer pieces, with the specific probe question randomly assigned across participants). The two displays each consisted of four singles and two to four pairs. The number of singles did not vary between trials or displays, and they were included for two reasons: first, to minimize the possibility that observers would assume that pieces always belonged in pairs, and second, to make the task of numerosity comparison more difficult. Thus, each display contained eight, 10, or 12 total pieces. On each trial, single pieces were chosen randomly from all possible pieces with replacement.

One display always contained blue pieces (RGB: 72, 160, 248), and the other orange (RGB: 234, 155, 86). Each participant was randomly assigned to always see the blue display or the orange display first. All puzzle-piece stimuli were initially embedded on a white background within 1,002 px × 374 px images, which were scaled down and randomly positioned in the display. Each image was randomly assigned a size between 105 px and 145 px width (height fixed to the ratio of the source images) and oriented at a random angle between 0 and 360°, utilizing a 3 × 4 grid to prevent overlap among stimuli (with each item randomly jittered by 5 pixels horizontally/vertically). “Pair” images contained two pieces, with one on each side of the image. The sizes, positions, and orientations of the pieces in each pair were fixed to one another. “Single” images contained only one piece, occupying one side of the image with blank space on the other. This ensured that individual puzzle pieces (whether from images of pairs or singles) would not inadvertently appear in the space immediately across from the individual piece in images of singles when these images were randomly positioned in the display.

Crucially, the displays were split into two types: “matched” and “mismatched.” In “matched” displays, each of the pairs in the display was matched, and therefore could be combined into a complete object. In “mismatched” displays, each of the pairs in the display was mismatched, and therefore could not possibly be combined into a complete object. Each trial consisted of one matched display and one mismatched display.

The possible ratios between the two displays varied such that the number of pieces in the matched display could be equal to the number of pieces in the mismatched display (ratios of 8/8, 10/10, or 12/12), or unequal (ratios of 8/10, 10/12, 8/12, 12/10, 10/8, or 12/8). We included eight trials of each of the unequal ratio types. We included 16 trials of each equal-ratio trial type in order to obtain more precise data on the effect of “matchness” when the number of pieces did not actually differ across displays. The order of the matched and mismatched display for each trial (matched first or matched second) was also counterbalanced for each ratio. There were 96 trials in total, with trial order randomized.

### Analysis

In accordance with our preregistered analysis plan, participants were excluded if they failed to perform at or above 70% accuracy, or if they failed to contribute a complete dataset. There were 177 participants included in the analyses after these exclusions.^1^

In this and subsequent experiments, we conducted two types of analyses (both preregistered). First, we calculated the proportion of trials for each participant in which they chose the matched display as being less numerous than the mismatched display, and performed a one-sample *t* test across participant means, comparing the means to 50% (chance). Significant above-chance responses here would indicate that matched displays were overall judged to be less numerous than mismatched displays.

To complement this simpler analysis, we also performed a classic psychometric analysis that is standard in the literature on numerosity estimation (e.g., Odic et al., 2013a, 2013b; Sanford et al., 2023). This analysis allowed us to more precisely model the relationship between participants’ numerosity judgments and the actual numerosity ratio between the matched and mismatched displays. We fit separate psychometric functions for each participant to determine the point of subjective equality (PSE) for matched and mismatched trials, where the PSE is defined as the numerosity ratio of matched and mismatched displays at which the numerosity of the two displays appears to be equivalent (see details on fitting procedure in next paragraph). We then performed a one-sample *t* test on the shift in the mean PSE across participants relative to baseline, where the baseline corresponds to a numerosity ratio of one (i.e., the numerosity of the two displays is seen as equivalent when there are an equal number of pieces in each one). A PSE lower than one would indicate that a smaller number of mismatched pieces is required (relative to the number of matched pieces) to make the number of pieces in the mismatched and matched displays appear equivalent.

The psychometric function for each participant was fit to individual trial-level data using the following procedure. First, the numerosity ratio, *r*, was determined for each trial. This *r* parameter was defined using the following rules:

$$\begin{array}{l}\text{If } {n}_{\mathrm{mismatched}}>{n}_{\mathrm{matched}}: r={n}_{\mathrm{mismatched}}/{n}_{\mathrm{matched}} \\ \text{If }{n}_{\mathrm{matched}}>{n}_{\mathrm{mismatched}}: r=2-{n}_{\mathrm{matched}}/{n}_{\mathrm{mismatched}} \\ \text{If } {n}_{\mathrm{mismatched}}={n}_{\mathrm{matched}}: r=1\end{array}$$

The parameter was defined in this way in order to ensure that the ratios reflected symmetrically around 1: 0.50, 0.75, 0.80, 1, 1.20, 1.25, and 1.50.

Next, we modeled the response probability at each numerosity ratio. Trials in which participants responded that the matched display had fewer pieces were coded as 1, and other trials were coded as 0. Using the cumulative normal distribution in R (*pnorm*) and maximum likelihood estimation, we fit three parameters for each participant: a lapse parameter (*g*), a Weber fraction (*w*), and a shift parameter (*d*). These parameters are illustrated in the following piecewise equation, where *p* represents the probability that a participant would respond that the matched display has fewer pieces for a given numerosity ratio:

$$p=\left\{\begin{array}{ll}\left(1-g\right)*\left[1-\frac{1}{2}erfc\left(\frac{\left(r-d\right)-1}{\omega \sqrt{2}\sqrt{{1+\left(r-d\right)}^{2}}}\right)\right]+\frac{g}{2},& r>\left(1+d\right)\\ \left(1-g\right)*\left[1-\left[1-\frac{1}{2}erfc\left(\frac{\left(2-\left(r-d\right)\right)-1}{\omega \sqrt{2}\sqrt{{1+\left(2-\left(r-d\right)\right)}^{2}}}\right)\right]\right]+\frac{g}{2},& r<\left(1+d\right)\end{array}\right.$$

The lapse parameter (*g*) corresponds to the proportion of guesses. The Weber fraction (*w*) corresponds to the steepness of the psychometric function. The shift parameter (*d)* was the measure of interest: It corresponds to the horizontal shift of the psychometric function and thus represents the “bias” away from a PSE (point of subjective equality) of 1. If matched displays and mismatched displays with the same number of pieces appear to be equally numerous, we would expect a PSE of 1. However, if participants perceive matched displays as less numerous than equinumerous mismatched displays—in line with our prediction—we would expect a PSE shifted below one, corresponding to a negative *d*.

### Results

We predicted that participants would more often respond that matched displays had fewer pieces (or, correspondingly, that mismatched displays had more pieces). These predictions were confirmed: participants tended to report that matched displays were less numerous more often than mismatched displays (*M* = 51.45%), *t*(176) = 4.53, *p* < .001, *d*_*z*_ = 0.34, 95% CI = [50.82%, 52.07%] (Fig. 3A). This effect was also evident nonparametrically: 118 of 177 participants responded in this direction in at least 50% of trials. The secondary analysis on psychometric function fits confirmed these results: the mean PSE across participants was significantly lower than one (*M* = 0.992), *t*(176) = 3.04, *p* = .003, *d*_*z*_ = 0.23, 95% CI = [0.987, 0.997]. This suggests that in order for participants to be equally likely to answer that matched and mismatched displays had fewer pieces, the ratio of mismatched to matched pieces had to be lower, more broadly indicating that matched displays appeared less numerous to participants. No significant difference was observed in the mean proportion of matched-display responses between participants who were asked about which display had more pieces vs. those asked about which display had fewer pieces (92 vs. 85); unpaired *t* test: *t*(172.43) = 0.55, *p* = .58.

These results suggest that estimates of numerosity were sensitive to the combinability of object parts (here, puzzle pieces)—as if the visual system was automatically combining discrete parts into “wholes” and comparing numerosities based on these combined items, rather than on the actual pieces themselves.

## Experiment 2: Which had more “blickets”?

We have suggested that the present effects arise because the mind estimates numerosities over whole objects—including possible but nonexistent objects mentally represented by virtue of their combinable parts. However, perhaps our previous results might be explained by features of the task itself. In particular, the instructions may have been sufficiently vague that they were open to misinterpretation. Participants were instructed to report which display had more or fewer pieces but were not explicitly told what was meant by “piece.” It is possible that some participants interpreted “piece” to mean any object—including those objects that could be formed by combining two smaller pieces—rather than interpreting “piece” as the puzzle pieces themselves. Experiment 2 addressed this possibility by introducing an extended instruction phase that included an explicit training phase (with feedback) about what counted as a piece, referred to as “blickets” in this new experiment. Training participants with such labels ensured that participants would interpret the individual pieces as the units to be enumerated (Brooks et al., 2011).

### Methods

#### Participants

Two-hundred participants were recruited from Prolific. We chose this sample size to match that of Experiment 1.

#### Stimuli, procedure, and analyses

The stimuli for the current experiment were identical to those from Experiment 2 (Fig. 3B). The procedure was the same as that of the previous experiment, with several changes introduced to ensure that participants unambiguously understood that they were to compare the number of individual puzzle pieces across displays. First, rather than referring to the stimuli as “pieces,” we referred to them as “blickets,” and participants were explicitly told that each individual puzzle piece counted as a blicket. We provided several introductory displays in which participants were told how many blickets were in the display (e.g., “Here are 6 blickets…,” “Here are 8 blickets…”). Next, participants completed a qualifying preexperiment task, in which they were asked to indicate the exact number of blickets in five sample displays (similar to the displays that would be presented during the main task). Participants could only proceed to the next sample display if they correctly entered the number of pieces in the display. If participants made three errors, they were not permitted to complete the main experiment and were redirected to the Prolific site. If they passed this qualifying task, participants were permitted to continue on to the main experiment, which was identical to Experiment 1 except that pieces were referred to as “blickets.” Analysis procedures were the same as in Experiment 1.

### Results

The criteria for exclusion were preregistered, and were the same as in Experiment 1, with the additional criterion that a participant was excluded if they made three errors in the preexperiment instructions/training task. This left 172 participants. Our first analysis was consistent with the results of Experiment 1: participants tended to judge the matched displays to be less numerous more often than the mismatched displays (*M* = 51.47%), *t*(171) = 4.90, *p* < .001, *d*_*z*_ = 0.37, 95% CI = [50.88%, 52.06%] (Fig. 3B). This effect was also evident nonparametrically: 118 of 172 participants responded in this direction in at least 50% of trials. The secondary psychometric analysis showed similar results: the mean PSE across participants was shifted to be significantly below baseline (*M* = 0.992), *t*(171) = 3.98, *p* < .001,* d*_*z*_ = 0.30, 95% CI = [0.988, 0.996]. No significant difference was observed in the mean proportion of matched-display responses between participants who were asked about which display had more pieces vs. those asked about which display had fewer pieces (87 vs. 85); unpaired *t* test: *t*(169.79) = 0.60, *p* = .55.

Taken together, these results suggest that the effect of combinability on numerosity estimation was not driven by ambiguity in the task instructions. Instead, even when participants *knew* that individual segmented objects were the units to be compared across displays, they could not resist the tendency to treat the discrete wholes formed by combinable object parts as the units for visual numerosity estimation.

## Experiment 3: Combinability per se?

Although previous experiments identified an influence of combinability on numerosity, it remains unclear whether this effect can be extricated from lower-level spatial grouping cues—in particular, modal completion (Wagemans et al., 2012). Specifically, it is possible that the paired pieces were modally completed at their top and bottom contours, giving rise to a percept of a single object without a need to invoke part–whole relationships. This alternative explanation is important given evidence that both modal completion and spatial alignment affect numerosity (e.g., Adriano et al., 2021; DeWind et al., 2020). Notably, if modal completion were solely responsible, it should influence both matched and mismatched pairs, although it is still possible that it might do so more strongly in the matched pairs since their aligned indentations and protrusions might facilitate modal completion. To address this possibility, we take inspiration from Guan and Firestone (2020), who found that the perception of object combinability persisted even when those objects were displaced such that their contours were misaligned. The present experiment asks whether the findings of the previous two experiments generalize when the pieces in stimulus pairs are displaced, thereby disrupting contour alignment and modal completion—and, consequently, testing whether combinability influences numerosity beyond these lower-level effects.

### Methods

#### Participants

Two-hundred participants were recruited from Prolific. We chose this sample size to match that of Experiments 1 and 2.

#### Stimuli, procedure, and analyses

We modified the paired stimuli (both matched and mismatched pairs) by vertically offsetting the left and right pieces in each pair from each other by 20% of the image height (half the height of the protrusions). This ensured that paired pieces still faced each other, but their outer contours and protrusions were misaligned (Fig. 3C). Otherwise, this experiment proceeded identically to Experiment 2.

### Results

The preregistered exclusion criteria were identical to Experiment 2, leaving 180 participants. As in Experiments 1 and 2, participants tended to judge the matched displays to be less numerous more often than the mismatched displays (*M* = 51.45%), *t*(179) = 4.75, *p* < .001, *d*_*z*_ = 0.35, 95% CI = [50.85%, 52.06%] (Fig. 3C). This effect was also evident nonparametrically: 122 of 180 participants responded in this direction in at least 50% of trials. The secondary psychometric analysis showed similar results: the mean PSE was shifted significantly below baseline (*M* = 0.990), *t*(179) = 4.74, *p* < .001,* d*_*z*_ = 0.35, 95% CI = [0.986, 0.994]. No significant difference was observed in the mean proportion of matched-display responses between participants who were asked about which display had more pieces vs. those asked about which display had fewer pieces (98 vs. 82); unpaired *t* test: *t*(167.97) = 0.20, *p* = .84.

Recall that our modifications to the stimuli were intended to disrupt modal completion. Notably, previous studies have shown that misaligning objects to disrupt illusory contour formation also diminishes their effect on numerosity judgments (Adriano et al., 2021). Therefore, the present results suggest that the perception of combinability per se influences numerosity estimation, above and beyond lower-level spatial linkages such as those induced by modal completion.

## Experiment 4: Generalization to a new combinability manipulation

The previous three experiments used the same “combinability” manipulation between matched and mismatched displays. However, manipulating the type of fit (i.e., whether the protrusions match the indentations) is not the only way that pieces can be made more or less combinable. Consider Fig. 3D. Although the matching pieces can easily combine into one discrete object, when they are each rotated 180° to face away from one another, they can no longer easily combine. In a final experiment, we ask whether the effect of combinability on visual numerosity estimation would generalize to a completely different combinability manipulation.

### Methods

#### Participants

Two-hundred participants were recruited from Prolific. We chose this sample size to match the 200 used in each of the previous three experiments.

#### Stimuli and procedure

In line with the previous three experiments, stimuli consisted of the same set of “puzzle pieces.” However, unlike the prior experiments, paired pieces *always* had matching indentations and protrusions. Rather than categorizing pairs as “matched” or “mismatched,” pairs were either “inward” (indentations and protrusions oriented *towards* each other) or “outward” (indentations and protrusions oriented *away* from each other; Fig. 3D). Inward pairs represented easily combinable pieces of the exact kind featured in the “matched” pairs of Experiments 1–3. By contrast, outward pairs could not be easily combined into one object, as doing so would involve mentally rotating one of the two pieces in a pair by 180°. Because the protrusions and indentations were always matched, any observed differences between inward and outward displays can be attributable to the difference in orientation rather than peculiarities of the puzzle-piece parts. The procedure and analyses were identical to that of Experiments 2 and 3.

### Results

The criteria for exclusion were the same as in Experiments 2 and 3 and were preregistered. This left 177 participants. As expected, participants tended to report that inward displays were less numerous than outward displays (*M* = 59.13%), *t*(176) = 17.67, *p* < .001, *d*_*z*_ = 1.33, CI = [58.11%, 60.14%] (Fig. 3D). This effect was also evident nonparametrically: 162 of 177 participants responded in this direction in at least 50% of trials. The secondary psychometric analysis showed similar results: the mean PSE across participants was also significantly shifted below baseline (*M* = 0.935), *t*(176) = 15.38, *p* < .001, *d*_*z*_ = 1.16, CI = [0.927, 0.944]. No significant difference was observed in the mean proportion of matched-display responses between participants who were asked about which display had more pieces vs. those asked about which display had fewer pieces (106 vs. 71); unpaired *t* test: *t*(170.76) = 0.73, *p* = .46.

Interestingly, the combinability effects in the current experiment appeared to be stronger than in the previous experiments: participants judged the inward displays to be less numerous about 59.1% of the time, compared to about 51.5% of the time for Experiments 1–3. This was confirmed statistically: exploratory unpaired *t* tests showed that the current experiment’s combinability effect (proportion of trials where combinable displays were chosen to be less numerous) was significantly greater than the effect in the other three experiments (all *t* values > 9.11, *p* values < .001), while the other three experiments did not differ significantly from one another (all *t* values < 1.25, *p* values > .60; *p* values corrected for six comparisons using the Bonferroni–Holm method).

These results suggest that the phenomenon observed in Experiments 1–3 is generalizable to a different form of visual combinability—specifically, the effect of piece orientation. Even though all the pairs in this experiment had matching protrusions and indentations, participants dramatically underestimated the number of pieces in inward displays relative to outward displays. Taken together, these results provide additional evidence that the visual system tends to “enumerate” combinable parts in terms of their distinct wholes.

## General discussion

Humans have the impressive ability to estimate numerosities, a capacity that arises early and develops with age (Izard, et al., 2008, 2014; Xu & Spelke, 2000). Some research suggests that this capacity may even display telltale signatures of automatic visual processing, such as adaptation (Burr & Ross, 2008; Myers et al., 2025; cf. Yousif et al., 2024). The present experiments used combinable objects to ask what may be treated as perceptual “units” in visual numerosity estimation. Across four experiments, we found that displays with combinable pieces were reported as less numerous than displays with noncombinable pieces. This effect was observed for matched vs. mismatched pairs of object parts (Experiment 1), persisted even when participants were instructed to treat individual pieces as the units to be enumerated (Experiment 2), could not be explained solely by lower-level influences such as modal completion (Experiment 3), and generalized to a different combinability manipulation using inward-facing vs. outward-facing pairs (Experiment 4). Taken together, these findings suggest that the mind automatically considers the potential wholes that combinable parts can form—and in turn treats these composite objects as the inputs to visual numerosity estimation. More broadly, the current studies extend previous work on the perception of “possible objects” beyond recognition (Guan & Firestone, 2020) to its downstream effects on other mental processes.

### Combinability and grouping

Notably, the existence of part–whole effects in visual numerosity estimation was not a foregone conclusion. On one hand, general grouping effects have been clearly established in numerosity tasks—whether through explicit or modally completed connections (e.g., Adriano et al., 2021; Franconeri et al, 2009; Qu et al., 2024); lower-level features such as spacing, color, contrast, or orientation (Ciccione & Dehaene, 2020; DeWind et al., 2020; Lei, & Reeves, 2023; Qu et al., 2022; Zhao & Yu, 2016); or higher-level properties such as the typical arrangement of objects in a scene (e.g., chairs facing a table; Carter & Kaiser, 2024). On the other hand, previous work had not yet established if and how part–whole effects might play a role in visual numerosity estimation. Moreover, our effects went beyond classic Gestalt grouping cues such as parallelism and even modal completion Wagemans et al. (2012). In particular, while the strength of such cues in Experiments 1 and 2 might have differed between matched and mismatched pairs where the protrusions met the indentations (Figs. 3A and 3B), this was not the case in Experiment 3 where pieces were vertically misaligned (Fig. 3C), and yet part–whole effects of the same magnitude were still observed. It is also worth noting that these grouping cues do not apply in the case of Experiment 4, and yet the effect was stronger in that case. Thus, our findings may be construed as a novel influence of high-level grouping (here, part–whole combinability) on visual number estimation.

It is worth acknowledging that the absolute size of the effects observed in our studies is smaller than that observed in previous work. For example, in Franconeri et al. (2009), participants underestimated number by almost 25% in the barbell condition. In our work, by contrast, participants judged combinable displays as containing more items than equinumerous noncombinable displays about 51.5% of the time (Experiments 1–3) or 62% of the time (Experiment 4). However, the magnitude of the effects reported here is unsurprising given the subtlety of our combinability manipulation, and it is in line with relatively small effect sizes for other subtle manipulations such as those producing modally completed connectors (Adriano et al., 2021).

Importantly, neither the part–whole combinability studied here, nor explicit connections or other grouping cues (e.g., as in Franconeri et al., 2009), have “all-or-none” effects on perceived number. In other words, connecting pairs of items does not strictly halve perceived number. While our data (and, to our knowledge, previous work) cannot definitively explain why, one possibility is that grouping or combinability cues operate probabilistically: their presence increases the likelihood—but does not guarantee—that certain pairs of items will be treated as a single unit during enumeration. Another possibility is that enumeration and grouping or combinability processes run in parallel, such that some items may be enumerated before grouping effects fully take hold. Future work can investigate these possibilities more directly.

### The nature of numerosities in visual number estimation

While the theoretical status of the ANS remains a subject of intense discussion (e.g.,, Clarke & Beck, 2021; Gebuis et al., 2016; Leibovitch et al., 2017), our studies speak to current debates over what *kinds* of number such a system might represent—natural numbers (e.g., 8, 9, …), rational numbers (e.g., 8.5, 9.0, …), irrational numbers (e.g., √2, π, …)—or even whether it represents number at all. Recently, Clarke and Beck (2021) argued that the system for estimating numerosities represents both natural and nonnatural rational numbers (i.e., that it is a system for approximating *number* per se). However, some researchers suggest that such a system might instead represent nonnumerical features such as area, convex hull, or other such properties that covary with number. Indeed, participants routinely underestimate numerosities when objects are smaller or have less perimeter (Gebuis et al., 2016; Leibovich et al., 2017). Our studies suggest a different possibility: that in some cases, the system for estimating numerosities represents object parts as such (e.g., nine half-objects as a numerosity of 4.5)—that is, in terms of a rational number value, just as Clarke and Beck suggest (see also Yousif, 2021). Indeed, if observers assign a rational number value to pairs of half-objects, then that would give rise to the kinds of results we have observed. While such an explanation would still need to account for effects of combinability (i.e., when object parts would and would not be treated as wholes), object parts of the kind used in our studies could prove useful in future research addressing what kind(s) of number the system for processing numerosities represents.

### “Fitting” relations in perception and cognition

Our work contributes to the growing literature showing that automatic visual processing extracts not only basic visual properties such as color, texture, shape, or location, but also relations between objects (for reviews and commentaries, see Hafri & Firestone, 2021; Hafri et al., 2023; Hafri & Papeo, 2025). While the primary goal of the current studies was to determine how potential part–whole relations influence downstream visual processes like number estimation, the combinability of our matched puzzle pieces can also be construed as instantiating a *fitting* relation (unlike the mismatched pieces, which cannot). Interestingly, this kind of fitting—between two tightly fitting object parts—bears similarities to the notion of *tight-fit* that has been explored both developmentally and cross-linguistically (Bowerman, 1996; Hespos & Spelke, 2004; Johannes et al., 2016; Landau et al., 2017; Levinson, 2003). For example, Korean lexicalizes such tight-fitting (*“kkita”*; e.g., a key fitting snugly into a lock) in ways that distinguish it from more general notions of containment (*“nehta”*; e.g., a pen loosely placed in a mug). While it remains debated whether such distinctions persist cognitively into adulthood for speakers of languages that do not explicitly lexicalize them (Norbury et al., 2008; Landau et al., 2023), our results could be considered complementary perceptual evidence for this cognitive distinction. We speculate that such *tight-fitting* relations might indeed be privileged in visual processing precisely because they transform separate entities into a unified whole (unlike related notions such as containment, which may keep the participating entities as distinct perceptual objects). Future work could provide additional perceptual evidence for this distinction by directly comparing perceptual effects for the kind of *fitting* relation studied here (involving tightly fitting object parts) and others (e.g., a knife loosely placed in a cup; Hafri et al., 2024).

### Looking forward

Our studies raise many exciting questions for future research. For example, what are the limits on how the visual system processes geometric fit? In particular, is there a “capacity limit” on how many pieces can be simultaneously combined? In the current study, combinable stimuli were limited to pairs of pieces. However, future research could investigate whether larger groups of pieces are represented as the single whole they could create, and whether this combinability would continue to bias the downstream effects on numerosity estimation (as in Fig. 1C). With an increasing number of pieces in each group, perhaps the visual system takes longer to combine them, or completely fails to do so—consistent with the three- or four-item limit in studies of object-based attention (Pylyshyn & Storm, 1988; Scholl, 2001).

A second open question is what other processes might be influenced by combinability. One example to consider is object-based attention (Chen, 2012; Scholl, 2001). For example, previous research has identified a “same-object advantage,” in which observers respond faster to a target when it appears within a cued object than in a noncued object at an equal distance (Egly et al., 1994; cf. Chou & Yeh, 2018). Our findings raise the intriguing possibility of a “combinable-objects advantage,” in which attention would spread more easily between two locations on combinable pieces compared with locations on noncombinable pieces. Another example to consider is object-based warping, in which the distance between two dots within an object is perceived as greater compared to the same distance across two different objects (Vickery & Chun, 2010). Perhaps the distance between dots on two combinable pieces might likewise be perceived as greater compared to when they are on noncombinable pieces.

More broadly, our results suggest that the visual system treats combinable parts as a whole object when estimating numerosity from a visual display. In other words: possible objects “count” too.

## Data Availability

All experiment materials, including preregistrations, stimuli, code, analyses, and anonymized data, are available at: https://www.palresearch.org/partwholenumber

## Footnotes

1 It is worth noting that none of the reported effects depended on this exact exclusion criterion. When we ran exploratory analyses excluding only participants with below chance accuracy (<50%), all effects remained statistically significant and in the same direction across experiments. (The sole exception was the psychometric analysis in Experiment 2, which—while not statistically significant—trended numerically in the same direction.)

## Code availability

See “Data Availability” above
